# Supplementary material for: Accuracy of Fibrosis-4 Index in Identification of Patients with Cirrhosis Who Could Potentially Avoid Variceal Screening Endoscopy
Source: J Clin Med. 2020 Oct 29;9(11):3510. doi: 10.3390/jcm9113510 (PMC7692323; doi:10.3390/jcm9113510)
Supplement: Supplementary file 1 [file jcm-09-03510-s001.pdf]

Supplemental figure 1

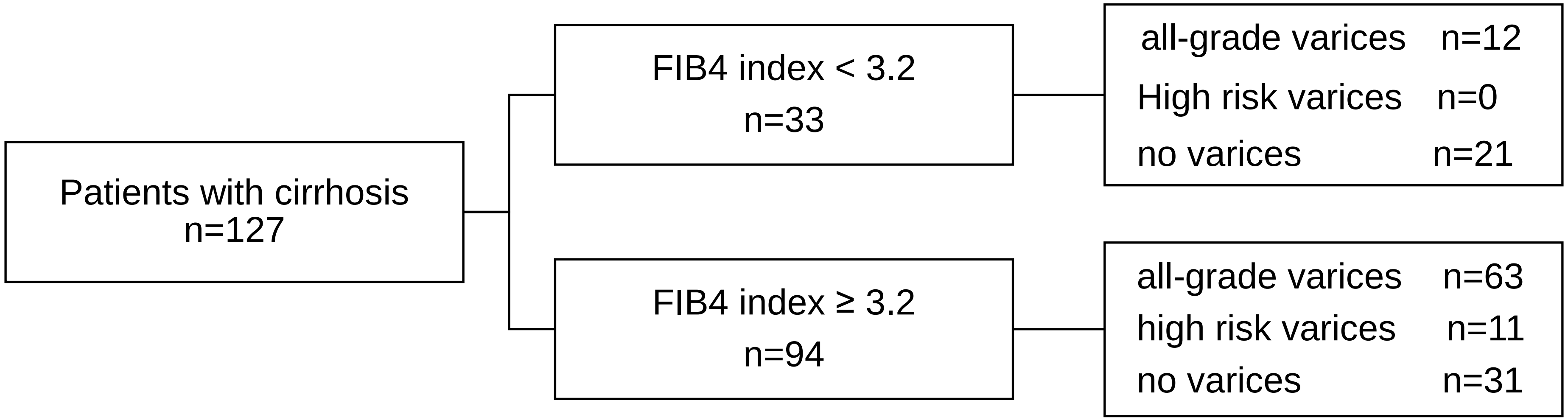

Supplemental figure 2

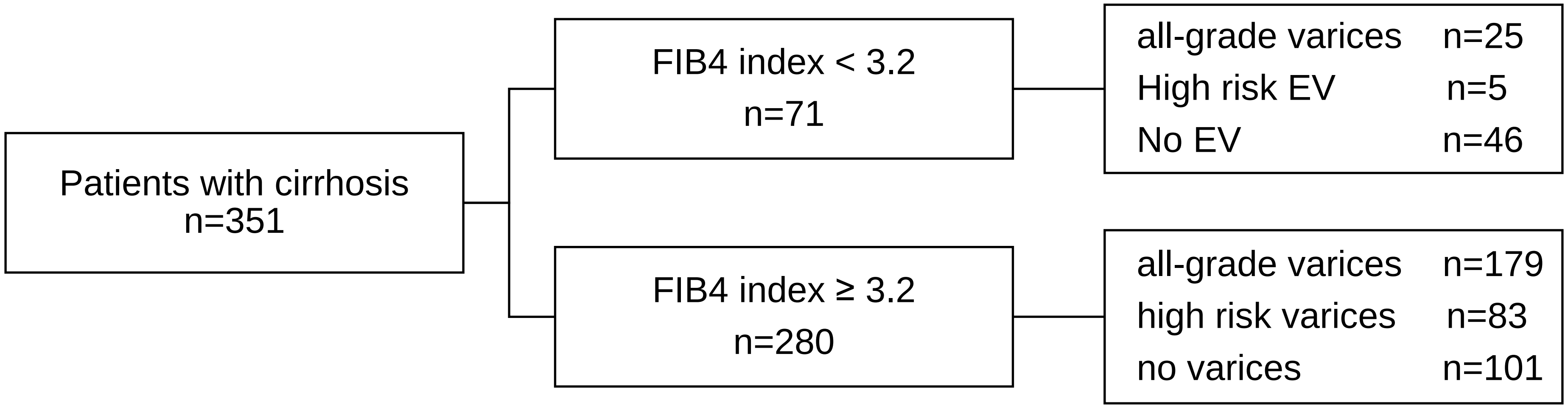

Supplemental table 1 Performance of fibrosis indices for the prediction of high-risk varices in the estimation set (n=127)

Fibrosis indices	Cut-off	AUROC	Se	Sp	PPV	NPV
**FIB4-index**	3.20	0.53	1.0	0.28	0.12	1.00
**Hyaluronic acid (ng/mL)**	181.32	0.49	0.91	0.28	0.11	0.97
**Platelet (x10^3/μL)**	11.90	0.59	0.82	0.47	0.13	0.97
**ELF score**	10.53	0.48	0.91	0.21	0.10	0.96
**7S collagen (ng/mL)**	5.00	0.53	0.91	0.24	0.10	0.97
**APRI**	1.16	0.56	0.73	0.53	0.13	0.95
**M2BPGi (COI)**	3.96	0.53	0.73	0.44	0.11	0.94
**PIIINP (ng/mL)**	3.90	0.48	0.09	1.00	1.00	0.92
**TIMP-1 (ng/mL)**	227.2	0.57	0.67	0.12	0.06	0.78

Se, sensitivity; Sp, specificity; PPV, positive predictive value; NPV, negative predictive value; Fib-4 index, the fibrosis index based on four factors; ELF score, enhances liver fibrosis score; 7S collagen, 7S fragment of type 4 collagen; APRI, the aspartate aminotransferase-to-platelet ratio index; M2BPGi, mac-2-binding protein glycosylation isomer; PIIINP, type 3 procollagen-N-peptide; TIMP-1,tissue inhibitor of metalloproteinase 1.

Supplemental table 2. Performance of a FIB-4 cut-off of 3.20 for the prediction of high-risk varices in the validation cohort (n=351)

Validation set (n=351)	High-risk varices			
	Se	Sp	PPV	NPV
Fib4-index (cut-off 3.20)	0.94	0.25	0.30	0.93

Fib-4, the fibrosis index based on four factors; Se, sensitivity; Sp, specificity; PPV, positive predictive value; NPV, negative predictive value

Supplemental Table3 Multivariate analysis predicting the presence of high risky varices in patients with cirrhosis, by logistic regression method

	Odds ratio(95%CI)	P value
FIB-4 index	0.003250 (0.000453-3.28)	0.993
Hyaluronic acid	0.413 (0.0145-11.8)	0.605
Platelet	0.299 (0.0481-1.85)	0.195
ELF score	0.548 (0.105-2.86)	0.475
7S collagen	3.73 (0.271-51.5)	0.325
APRI	0.924 (0.141-6.06)	0.935
M2BPGi	0.405 (0.0289-5.68)	0.502
PIIINP	0.446 (0.0367-5.42)	0.526
TIMP-1	1.83 (0.349-9.59)	0.475

Fib-4 index, the fibrosis index based on four factors; ELF score, enhances liver fibrosis score; 7S collagen, 7S fragment of type 4 collagen; APRI, the aspartate aminotransferase-to-platelet ratio index; M2BPGi, mac-2-binding protein glycosylation isomer; PIIINP, type 3 procollagen-N-peptide; TIMP-1,tissue inhibitor of metalloproteinase 1.

For reviewer only The cutoff values for the FIB4 indexes in the validation cohort (n=351)

	F0	F1	F2-3
Fib4-index≧2.847	110	101	88
Fib4-index＜2.847	37	15	0
	F0	F1	F2-3
Fib4-index≧3.058	102	97	87
Fib4-index＜3.058	46	18	1
	F0	F1	F2-3
Fib4-index≧3.089	102	97	86
Fib4-index＜3.089	46	18	2
	F0	F1	F2-3
Fib4-index≧3.172	101	96	85
Fib4-index＜3.172	47	19	3
	F0	F1	F2-3
Fib4-index≧3.185	101	96	84
Fib4-index＜3.185	47	19	4
